# Effects of flower essences on nursing students’ stress symptoms: a randomized clinical trial*

**DOI:** 10.1590/1980-220X-REEUSP-2021-0307

**Published:** 2022-01-05

**Authors:** Lucia Maria Nunes Freire de Albuquerque, Ruth Natalia Teresa Turrini

**Affiliations:** 1 Universidade de São Paulo, Escola de Enfermagem, Programa de Pós-Graduação Enfermagem na Saúde do Adulto, São Paulo, SP, Brazil.; 2 Universidade de São Paulo, Escola de Enfermagem, Departamento de Enfermagem Médico-Cirúrgica, São Paulo, SP, Brazil.

**Keywords:** Education, Nursing, Baccalaureate, Students, Nursing, Psychological Distress, Flower Essences, Complementary Therapies, Bachillerato en Enfermería, Estudiantes de Enfermería, Distrés Psicológico, Esencias Florales, Terapias Complementarias, Bacharelado em Enfermagem, Estudantes de Enfermagem, Angústia Psicológica, Essências Florais, Terapias Complementares

## Abstract

**Objective:**

To analyze the effects of flower essence bouquets on the signs and symptoms of stress in nursing students.

**Method:**

Randomized clinical trial, triple blind, with two groups, flower essence group and placebo group, carried out with 101 nursing students. The groups used the formula for 60 days at a dosage of 4 drops 4 times a day. The outcome was evaluated using the Baccaro Test and the Perceived Stress Scale applied at the beginning and at the end of the intervention. The outcome analysis was performed using the mixed effects model, with 〈 = 5% and the effect size verified by the Cohen’s d test.

**Results:**

There was no significant difference between the groups in stress reduction (p > 0.05). Both groups showed a reduction in scale scores (p < 0.001) with large effect size. There was an influence of the COVID-19 pandemic in the reduction of Baccaro Test scores.

**Conclusion:**

The intervention with flower essence therapy was not more effective than placebo in reducing stress signs and symptoms. Brazilian Registry of Clinical Trials: UTN U1111–1257-5715.

## RESUMO

**Objetivo:**

Analisar os efeitos do buquê de essências florais nos sinais e sintomas do estresse em estudantes de enfermagem.

**Método:**

Ensaio clínico randomizado, triplo cego, com dois grupos, floral e placebo, realizado com 101 estudantes de enfermagem. Os grupos utilizaram a fórmula por 60 dias na dosagem de 4 gotas 4 vezes ao dia. O desfecho foi avaliado pelas escalas Teste de Baccaro e Escala de Estresse Percebido, aplicadas no início e no final da intervenção. A análise do desfecho foi feita pelo modelo de efeitos misto, com 〈 = 5% e o tamanho de efeito verificado pelo teste d-Cohen.

**Resultados:**

Não houve diferença significativa entre os grupos na redução do estresse (p > 0,05). Ambos os grupos tiveram redução nos escores das escalas (p < 0,001) com tamanho de efeito grande. Houve influência da pandemia COVID-19 na redução dos escores do Teste de Baccaro.

**Conclusão:**

A intervenção com terapia floral não foi mais efetiva que o placebo na redução de sinais e sintomas de estresse. Registro Brasileiro de Ensaios Clínicos: UTNU1111–1257–5715.

## RESUMEN

**Objetivo:**

Analizar los efectos del buqué de esencias florales en los señales y síntomas del estrés en estudiantes de enfermería.

**Método:**

Ensayo clínico randomizado, triple ciego, con dos grupos: floral y placebo realizado con 101 estudiantes de enfermería. Los grupos utilizaron la fórmula por 60 días con la dosis de 4 gotas 4 veces al día. El resultado se evaluó por las escalas: Prueba de Detección del Estrés (Baccaro) y Escala de Estrés Percibido aplicadas al principio y al final de la intervención. El análisis del resultado se realizó por el modelo de efectos misto, con 〈 = 5% y el tamaño de efecto averiguado por la d de Cohen.

**Resultados:**

No hubo diferencia significativa entre los grupos en la reducción del estrés (p > 0,05), ambos los grupos tuvieron reducción en los scores de las escalas (p < 0,001) con tamaño de efecto grande. Hubo influencia de la pandemia COVID-19 en la reducción de los scores de la Prueba de Detección del Estrés (Baccaro).

**Conclusión:**

La intervención con terapia floral no fue más efectiva que el placebo en la reducción de señales y síntomas de estrés. Registro Brasileño de Ensayos Clínicos: UTN U1111–1257–5715.

## INTRODUCTION

During the undergraduate course, nursing students present psychological and physiological manifestations of stress in greater proportion than students from other areas. Some situations can be considered stressful in nursing education, such as the contexts of practice, the differences between what they learn in theory and the reality they face, the curricular training, dealing with human limits (diseases/death), feelings of incapacity in face of the activities required, the quality of interpersonal relationships, the learning assessment processes, the intensive hours of classes, among others^(1)^.

A study that evaluated the stress of nursing students at a public university found that the percentage of undergraduates with physical symptoms (42%) was very close to those with psychological symptoms (45%), suggesting mixed vulnerability and somatization tendencies^(2)^. The academic stress perceived by Spanish students at the end of the first year of the nursing course included physical symptoms and signs such as headache, nail biting, muscle tension, tremors due to stress, diarrhea, and skin irritations. Furthermore, it was observed that 95% of female students had psychological symptoms, 92% physical symptoms, and 98% behavioral changes associated with the perception of stress^(3)^.

The concept of stress presents a theoretical progression that can be explained from three perspectives: in the biological repercussion of the phenomenon, in the stimulus that focuses on the psychosocial and social events that trigger the neurophysiological responses to stress, and a cognitivist perspective that assumes stress as a relationship between the individual and the environment^(4)^. It should be noted that stress helps the individual to face challenges and it should be seen as a negative event in clinical conditions of chronic stress.

The individual’s life history plays a fundamental role in how the student will react to different everyday stressors, along with factors associated with their education and academic experiences. Some practices, such as physical activities, sports, meditation, among others, have been reported and investigated as ways to relieve stress. A randomized controlled study carried out with undergraduate and graduate students in the United Kingdom found that the practice of mindfulness improved well-being during and after the exam period^(5)^.

It is assumed that other complementary health practices can contribute to the reduction of stress among students. Thus, the purpose of this study is to use flower essence therapy, a non-pharmacological intervention, for the homeostasis of the individual’s subtle energy, selecting flower essences aimed at reducing psycho-emotional symptoms resulting from stress. Flower essence therapy is included in the National Policy on Integrative and Complementary Practices at the Brazilian Public Health System and is recognized as a specialty by the Federal Nursing Council^(6)^.

The application of flower essences to specific emotions and attitudes was developed by an English physician in the 1930s and works through human energy fields, which in their turn influence spiritual, mental, emotional, and physical well-being. They help to comprehend the lessons of every illness, to face the challenges presented to our souls by emotional and physical pain and suffering^(7)^. Flower essence therapy helps in the process of expanding awareness by understanding the meaning of each life lesson, consequently a better understanding of the present moment and of the individuation process and finding oneself^(8)^.

The flower essences action mechanism can be explained by the quantum theory, which postulates that electromagnetic energy is not transmitted linearly, but in energy packets (quantum). The evolution in quantum knowledge allowed the proposition of concepts that contribute to a better understanding of the flower essence, understood as ‘in-formation’ available in the quantum field^(9)^. Living systems are considered complex, non-linear, with no thermodynamic equilibrium (change of state), self-organized on a holistic level according to the principles of quantum theory. The ‘in-formation’ carried by the floral formula favors quantum coherence and the assimilation of virtues by the individual. “This transmission takes place through a frequency of wave energy that carries the ‘in-formation’ into action, and activates cell membranes, leading to specific biochemical responses capable of changing the biological response that coordinates neurotransmitters, neuropeptides, and hormones, modulating the way one thinks, feels, and acts”^(9)^.

Stress reduction was observed in a placebo-controlled clinical trial with teachers from the basic education system who used Bach flower essences and the outcome was measured by the Stress Symptom List and by bioelectrographic aspects^(10)^. Research carried out with professors from a nursing course identified a 42.9% reduction in the level of occupational stress after using a formula containing Bach, California and Australian Bush flower essences^(11)^.

Studies that used flower essence therapy to reduce stress levels, despite having obtained beneficial results, showed methodological weaknesses for a clinical trial, mainly related to sample size and blinding. Thus, this study aimed to carry out a placebo-controlled and blinded clinical trial, without the incorporation of the emergency formula (composed of *Impatiens, Rock Rose, Clematis, Star of Bethlehem and Cherry Plum*), but with essences aimed at common stress symptoms. The study was guided by the research question: Can the flower essence formula consisting of *Cerato, Cherry Plum, Elm, Impatiens, Larch, Olive* and *White Chestnut* contribute to stress reduction in nursing students?

Therefore, the present study aimed to analyze the effects of flower essence bouquets on the symptoms and signs of stress in nursing students.

## METHOD

### Design and Local of Study

Randomized placebo-controlled, triple-blind clinical trial, with two arms, developed from September/2019 to February/2021 at the Nursing Laboratories Center for Teaching, Skills, Simulation and Research of the Nursing School of Universidade de São Paulo (EEUSP). Due to the COVID-19 pandemic, interaction with students was through electronic communication (email and Whatsapp) and by phone in 2020 and 2021.

### Participants

The sample consisted primarily of undergraduate nursing students at EEUSP and was complemented by students from another public and two private schools, regardless of the term of the course. Students who scored above 20 points on the Baccaro Test^(12)^, which characterizes a moderate to high level of stress, were include in the study. Students who used herbal medicines or medicinal plants, anti-depressants, anxiolytics, or some complementary practice were excluded. The loss of participants occurred when participants no longer responded to any telephone call, e-mail or cell phone message, and when they stated that they no longer wanted to participate in the research.

The sample could not be calculated using flower therapy studies as a reference, and although there was a study with professors which used the Baccaro Test, the sample was small (n = 14) with no control, and the scale analysis was carried out in an ordinal way with information that did not allow its use for a sample calculation. Therefore, in the absence of clinical trials with flower essence therapy for stress reduction with an adequate research design at the beginning of the study, an intervention study with massage and reiki for relief of signs and symptoms of stress in military personnel, as measured by the Stress Symptom List^(13)^, was used as a reference for the sample calculation. The selection of the study with reiki is explained by the fact that this intervention is a subtle-energy therapy similar to flower essence therapy. The effect size observed in this study was *f*= 0.396 and for a test power and confidence level of 95%, 102 individuals were required, divided in 51 in the Floral Group (FG) and 51 in the Placebo Group (PG).

Simple randomization with numbers generated in the software *Research Randomizer,* with 1:1 allocation was performed by the researcher herself and the drawing for allocation into groups was performed by the pharmacist responsible for preparing the formulas.

### Intervention

Bach’s flower essences C*erato (Ceratostigma wilimottianum)*, *Cherry Plum (Prunus cerasifera)*, *Elm (Ulmus procera)*, *Impatients (Impatiens glandulifera), Larch (Larix decidua), Olive (Olea europaea) and White Chestnut (Aesculus hippocastanum)* were selected by the researcher based on the experience of attending nursing students on flower essence therapy. The formulas were prepared in a 30 ml amber glass bottle with a perforated cap with a white seal and bulbs, and labeled according to randomization (Group 1 or Group 2). The floral formula consisted of 2 drops of the stock solution of each essence diluted in a water and 30% brandy solution and the placebo formula of water and 30% brandy. The groups used the respective formula for 60 days at a dosage of 4 drops 4 times a day, suggested to be taken when waking up, in the middle of the morning and the afternoon, and before bedtime. The solution bottles were provided by the researcher to the student, according to the randomization list. The dosage for using the formulas was checked at the meetings for participant monitoring.

### Outcomes

The primary outcome, the reduction of stress levels, was assessed by the Baccaro Test^(12)^ and by the Perceived Stress Scale^(14)^. The secondary outcome, students’ perception of the effects of the intervention, will not be the object of this publication.

### Collection Instruments

The sociodemographic and clinical questionnaire allowed the analysis of the variables: sex, age, children, occupational activity, place of residence, who the participant lived with, practice of sports and frequency, leisure activities, health problems, means of transportation to school, college financing, being a student at EEUSP, course term, and data collection during the pandemic.

The Baccaro Test is an adaptation of the Stress Symptom Checkup and contains 29 stress-related symptom items scored by a three-level Likert scale (0 to 2) for symptom frequency. No information about its validation was found and its score ranges from 0 to 58 points^(12)^.

The Perceived Stress Scale (PSS) measures the level at which individuals perceive stressful situations, that is, how unpredictable, uncontrollable, and overloaded the respondents assess their lives. The 14-item version was translated into Brazilian Portuguese and tested through internal consistency, the construct validity presenting adequate psychometric qualities^(14)^. Items are scored on a five-level Likert scale (0-4) and those with a positive connotation (4, 5, 6, 7, 9, 10 and 13) have their score reversed. The scale score is obtained by the sum of all items and can range from zero to 56^(14)^.

Coping strategies measured by the Brief-COPE scale were used as a moderating variable. The scale assesses the response to stressful situations and the way people deal with life problems, and consists of 14 sub-scales of two items each. The version translated and validated for Brazilian Portuguese presented reliability above 0.65 in the subscales, with most of them above 0.75^(15)^. Each subscale has its score and the higher the value obtained, the greater the use of a certain coping strategy.

### Recruitment

The research was disseminated through posters on boards and the TV in the main lobby of EEUSP, contact with class representatives, and a monthly disclosure email to students through the EEUSP communication department. To complete the sample size, participants from other schools were invited through oral disclosure among students, class representatives, and professors.

Interested parties got into contact by email or Whatsapp, and received an electronic form link to answer the sociodemographic-clinical questionnaire and the Baccaro Test (t_0_) to identify eligible participants. The introductory part of the electronic form contained information about the project and not submitting the form was interpreted as “not interested” in participating in the study.

### Masking

Participants, researchers, and statisticians accessed information about the designation of groups at the end of the study. The vials were prepared by the Pharmacy *Farmácia Néctar Homeopatia e Florais* and the pharmacist made the draw of the groups and identified the formulas.

### Data Collection Procedures

After identifying eligible students, they were randomized to flower therapy and placebo groups. Due to the COVID-19 pandemic and the determination of social isolation, it was necessary to change the collection procedure during the study. In the pre-pandemic collection (Sep – Dec/2019), an appointment was made for the delivery of the respective formula bottle (1 or 2) according to the list of randomization and guidelines for use, signature of the Free Informed Consent Form (FICF); in addition, the students received the electronic link to fill in the PSS (t_0_) and Brief-COPE (t_0_). After 30 days of use, by email or Whatsapp, a new appointment was made for the delivery of another bottle according to the respective group and the electronic link was sent to fill in the PSS (t_1_). After other 30 days (60 days after starting the use of the formula), the last in-person appointment was made by email or Whatsapp to close the survey with completion of the Baccaro Test (t_1_) and PSS (t_2_). During the pandemic (Jun/2020 – Feb/2021) the procedure was similar with the exception of in-person meetings, which were replaced by electronic or telephone communication. The formulas were sent to the participant’s home through the National Express Parcel Service (SEDEX) or by courier to ensure isolation.

### Statistical Analysis

Descriptive statistics was used to describe the variables; the chi-square test or Fisher’s exact test for qualitative variables for homogeneity of the groups or t-student test or Wilcoxon-Mann-Whitney test, depending on data normality, and when the variables dispersion was not similar, the Burnner Munzel test; the ANOVA mixed-effects model for comparison of groups and analysis of the interaction of the variables year of the course, student from USP, and pandemic in the outcome; Cohen’s d for effect size interpreted as small (0.20 – 0.49), medium (0.50 – 0.79), large (>0.80)^(16)^; reliability through Cronbach’s alpha. The analysis was performed by a statistician using the R software^®^ 4.0.4 and 5% significance level.

### Ethical Aspects

The project met the specifications of Resolution 466 of 2012, with approval by the Research Ethics Committee of EEUSP under opinion n° 3.342.181/2019. Participants filled out the FICF. There were no conflicts of interest related to the supplier of flower essences (Healing Flower Essences^®^) and the pharmacy that prepared the formulas. Placebo group participants who wished to received, at the end of the study, two vials of flower essences. Brazilian Registry of Clinical Trials: UTN U1111 – 1257-5715.

## RESULTS

**Figure 1.**
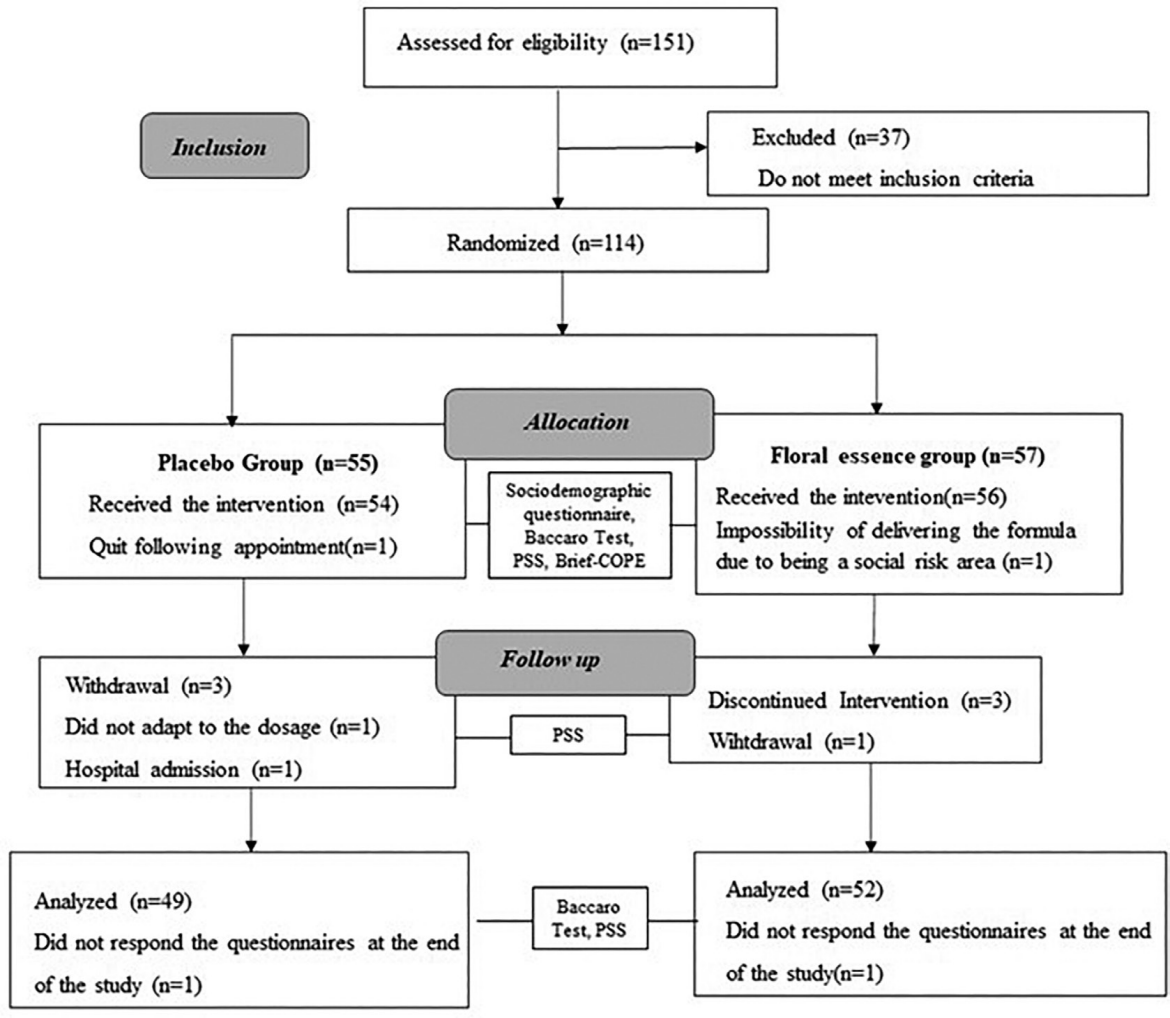
Clinical trial flow diagram. São Paulo, 2021.

Of the volunteers enrolled to participate in the study (n = 151), 37 (24.5%) were excluded because they did not reach the minimum score on the Baccaro Test (n = 29), were on use of antidepressant/anxiolytic medication (n = 6) or of another complementary practice (n = 2). Of the 114 eligible students, two dropped out before the first meeting and the others left the study throughout the follow-up, as shown in Figure 1.

It should be noted that due to the COVID-19 pandemic, for 50.5% (n = 51) of the students, the collection was carried out remotely. The distribution of students by study group was similar (0.767).

### Sociodemographic Characteristics

The sample consisted mainly of female, single, and EEUSP students (Table 1). The mean age was 22.1 (±4.1) in the PG and 22.3 (±5.4) in the FG with homogeneity of the groups.

**Table 1.** Distribution of nursing students according to sociodemographic-clinical variables and study group – São Paulo, Brazil, Sep/2019 to Feb/2021.

Variable	Categories	Placebo group		Flower essence group		p-value
		N	%	N	%	
Sex	Female	41	46.6	47	53.4	0.314*
	Male	8	61.5	5	38.5	
Marital status	Single	48	48.5	51	51.5	1.000**
	Married	1	50.0	1	50.0	
Children	Yes	1	100.0	–	–	0.485**
	No	48	48.0	52	52.0	
Type of school	Public	43	49.4	44	50.6	0.648*
	Private	6	42.9	8	57.1	
Student from USP	Yes	43	50.0	43	50.0	0.475*
	No	6	40.0	9	60.0	
Occupational activity	Yes	4	8.2	7	13.5	0.393*
	No	45	91.8	45	86.5	
Health problems	Yes	18	51.4	17	48.6	0.967*
	No	26	51.0	25	49.0	
Physical activity	Yes	17	53.1	15	46.9	0.658*
	No	27	48.2	29	51.6	

* chi-square test; ** Fisher test.

Of the students, 61.4% were from the city of São Paulo and 21.8% from the metropolitan region, the remaining from inland municipalities of the state and from other states in the Northeast and Southeast regions. The groups were homogeneous (p = 0.120) for the place of residence and 88% (n = 89) lived with their family (p = 0.541). Less than half of the students reported practicing some physical activity, with homogeneity between the groups (p = 0.658) and also for its frequency (p = 0.934), which was on average three times a week.

Most of them reported performing some leisure activity (89.1%; n = 90), with homogeneity in the groups (p = 0.393). According to the leisure classification used^(17)^, the main activity was the artistic one with the highest mention being watching movies, followed by the intellectual one, with higher frequency of reading. Numerically, there was a greater reference to touristic and physical activities in the FG.

About one third of the students reported a health problem, with gastritis/gastroesophageal reflux being the most reported problem in the PG (19.2%; n = 5) and atopic disorders in the FG (35.7%; n = 10). Of the students, 20.8% (n = 21) mentioned being in treatment and there was homogeneity between the groups (p = 0.926). The main treatments, except the use of contraceptives and those not specified, were consistent with the reported health problems.

Almost all students used some form of transportation to get to the university, except for one FG student who went to the university on foot. The mean number of means of transportation was 2.0 (±0.94) in the PG and 2.1 (±0.9) in the FG (p = 0.529), although up to five were reported.

Students from the first semester to the last term participated, but there was a prevalence of students from the second and sixth semesters in both groups (p = 0.499).

The Cronbach’s alpha reliability was 0.703 for the PSS, 0.502 for the Baccaro test and 0.595 for the Brief-COPE.

The groups were heterogeneous in terms of symptoms and stress perception, respectively, by the Baccaro Test (p = 0.040) and PSS (p = 0.039) and homogeneous by the Brief-COPE subscales. The coping strategies with the highest means were Planning (PG = 6.3% and FG = 6.3%), Self-blame (PG = 6.9% and FG = 6.7%) and Self-distraction (PG = 6.0% and FG = 6.2%).

### Intervention Outcome

The effect of flower essences in reducing the stress of nursing students could not be observed using the Baccaro Test and the PSS, as a reduction was observed in both groups throughout the study. Through Cohen’s d test, both groups and in both scales it was observed a large effect size (Table 2).

**Table 2.** Descriptive and variability measures for the Baccaro Test and Perceived Stress Scale before and after the intervention, according to the study group and Cohen’s d test – São Paulo, Brazil, Sep/2019 – Feb/2021.

Scale	Group	Time	N	Mean	SD	95%CI	p value time^1^	p value group: time^2^	Cohen’s d
Baccaro test	PG	t_0_	49	29.1	4.7	[27.8; 30.4]	0.001	0.319	1.17
	PG	t_2_	49	19.1	7.9	[17.0; 21.4]			
	FG	t_0_	52	27.6	5.0	[26.3; 29.0]			1.56
	FG	t_2_	52	19.5	8.6	[17.1; 21.7]			
PSS	PG	t_0_	49	38.9	4.3	[37.8; 40.2]	<0.001	0.256	1.32
	PG	t_1_	49	31.1	7.3	[29.1; 33.1]			
	PG	t_2_	49	28.0	8.4	[25.5; 30.2]			
	FG	t_0_	52	36.8	5.4	[35.4; 38.3]			1.65
	FG	t_1_	52	29.0	6.8	[27.2; 30.8]			
	FG	t_2_	52	28.0	7.8	[25.9; 30.1]			

^1^intragroup; ^2^ intergroups.

The analysis of the interaction of the variables year of the course, being a student at USP, and the COVID-19 pandemic with the outcome of the study showed that there was no interference in the results obtained by the PSS, but in the Baccaro test the variable pandemic interfered in the results of the scale (p = 0.046).

According to spontaneous reports, the formula was not used as recommended, especially in FG.

## DISCUSSION

The profile of nursing students was similar to a study carried out with nursing students in schools in the South and Southeast regions, where over 75% of them were single, without children, lived with their family, but the students’ mean age was higher^(18)^. Most students use two or more means of transportation to get to school, a situation that is consistent with the geographic extension of the city and the metropolis of São Paulo.

A little less than half of the students practiced some sport, at frequency similar to that found in Chilean university students^(19)^, but lower than observed in a study carried out with Spanish nursing students^(20)^.

Students reported health problems that configured as symptoms and signs of stress, with the most reported being atopic diseases, headache, and gastritis. Given the eligibility of students with high stress, the comparison with other studies can only be made with the post-intervention results, when the mean in both groups approached the mean score of 29.3 by the PSS in nursing students from a university in the state of São Paulo^(21)^. Among the main stress coping strategies used by students, only Planning was common to other studies^(22,23)^, as the others seem to be related to cultural and religious traditions of the countries.

The flower essence bouquet consisting of the essences *Impatiens, Cerato, Elm, White Chestnut, Olive, Cherry Plum, Larch* was not more effective than placebo in reducing signs and symptoms of stress as assessed by the Baccaro Test and PSS. The results of interventions with flower essence therapy differ according to the essence and measurement method adopted. A study with teachers from the basic education network also did not identify effects of flower essence therapy through PSS^(10)^. However, the use of flower essence therapy to control occupational stress in nursing professionals showed that 20% of participants who were under intense stress at the beginning of the study had their level of stress decreased to moderate after using flower essence therapy^(24)^.

The physician who developed the flower essences tried to show that health and illness are completely linked to the person’s way of life and to the need to change lifestyle, highlighting that they help in this learning and support the process of change^(7)^. The flower essences proposed for this study were intended to contribute to the increase in internal strength in times of weakness and to rescue the feeling of safety to face difficult tasks (*Elm*), mental tranquility and release of mental and emotional tensions (*Cherry Plum*), clarity of thought (*White Chestnut*), wisdom to understand and find individuality in decisions (*Cerato*), facing difficulties with confidence (*Larch*), inner calm to act in a balanced way in different situations of tension (*Impatiens*), and regeneration of physical and mental energy flow (*Olive*)^(7)^.

Studies evaluating the effect of individual essences are rare. Investigations have been carried out with the essence *White Chestnut*, as its effects extend to improved sleep and mental clarity. A Cuban study that used this essence observed its ability to suppress intrusive thoughts in apparently healthy adults with a different effect from the placebo group^(25)^. Since stress can compromise the quality of sleep of undergraduate students^(26)^ and the COVID-19 pandemic was associated with high levels of sleep disturbances and psychological distress in nursing students^(27)^, *White Chestnut* is a useful essence for these situations.

As flower essences act on the individual’s consciousness, they allow access to personal patterns, revealing aspects that can often be uncomfortable, a fact that can contribute to the slighter difference between PG and FG. When using the flower essence, a quantum connection is established between consciousness and ‘in-formation’ (plant consciousness) in the quantum field; however, any internal or external stimulus can break the coherence of this connection and the effectiveness of subtle treatment^(9)^. The journey to a new movement in life is permeated by resistance and discomfort because behaviors, thoughts, and emotions will be different from what the individual’s mind is used to experiencing^(9)^. In this process, the individual always has the free will to accept or reject the assimilation of a new virtue for a change.

It was observed that the pandemic phenomenon interfered in the stress results of students assessed by the Baccaro Test. The removal of stressors from academic daily life, such as the use of public transport, training and laboratory activities, in-person assessments, may have contributed to the reduction of stress regardless of the study group. However, the pandemic brought other stress-generating aspects. Assessment of emotional response and coping strategies used by nurses and nursing students during the COVID-19 pandemic observed that during the pandemic students experienced psychological stress, concern about their careers, excitement, and doubts^(28)^.

Although not evidenced in this study, perhaps due to the intervention, a study that evaluated the impact of the transition from in-person to remote classes due to the COVID-19 pandemic observed that 80% of students in the first term were anxious and stressed because of the effect of the pandemic on their academic education, mainly due to lack of ability to achieve personal goals and to deal with difficulties^(29)^. A more marked decrease in the mean stress measured by the PSS in the first terms of the undergraduate course can be expected, as academic stressors increase as the student enters into more specific content of care and in assistance activities with the ill individual^(30)^.

The placebo effect, considered an effect attributed to the expectation of cure, can be observed in any situation, for better or for worse^(9)^, because when an expectation is created, it changes the effect and consequently the emotional status. Furthermore, when participating in the treatment, the person feels cared for and this can lead to an improvement.

According to quantum theory, change and access to ‘in-formation’ present in the quantum field can be experienced by anyone as long as consciousness is open to this process. However, to achieve a significant effect it takes a lot of persistence, mentalization, and freedom from all negative thoughts. Therefore, placebo has some effect, but tends not to exceed 33% of the effects^(9)^.

Some limitations and biases were identified, such as the impossibility to control the use of the recommended dosage; change in stressors and data collection process which were not expected, due to the COVID-19 pandemic; exchange of information on the effect of formulas between groups by proximity and identification of vials as group 1 or 2; sample calculation that did not use a study with flower essences as reference; and heterogeneous groups in relation to the scales used.

## CONCLUSION

The intervention with flower essence therapy using the formula consisting of *Impatiens, Cerato, Elm, White Chestnut, Olive, Cherry Plum, Larch* was not more effective than placebo in reducing signs and symptoms of stress assessed by the Baccaro Test and the PSS. Despite the results obtained, it is not possible to consider the formula inadequate for stress reduction, due to the limitations presented in the study. Given the interference of the COVID-19 pandemic, the study has to be replicated with the control of some aspects of the student environment for better verification of the effect of the formula used.

## Financial support

This study was financed in part by the Coordenação de Aperfeiçoamento de Pessoal de Nível Superior – Brazil (CAPES) – Finance Code 001.

## References

[1] McCarthy B, Trace A, O’Donovan M, Brady-Nevin C, Murphy M, O’Shea M (2018). Nursing and midwifery students’ stress and coping during their
undergraduate education programs: An integrative review. Nurse Educ Today..

[2] Kestenberg CCF, Rosa BMS, Silva AV, Fabri JMG, Regazi ICR (2017). Stress in undergraduate nursing students. Rev Enferm UERJ..

[3] Encina RE, Meza LB, Auchter M (2018). Estres academico percibido por los estudiantes que finalizan el
primer año de licenciatura en enfermeria de la UNNE. Notas Enferm..

[4] Faro A, Pereira ME (2013). Estresse: revisão narrativa da evolução conceitual, perspectivas
teóricas e metodológicas. Psicol Saúde Doenças.

[5] Galante J, Dufour G, Vainre M, Wagner AP, Stochl J, Benton A (2018). A mindfulness-based intervention to increase resilience to stress
in university students (the Mindful Student Study): a pragmatic controlled
trial. Lancet Public Health..

[6] Conselho Federal de Enfermagem Resolução n° 581, de 19 de julho de
2018 (2018). Atualiza no âmbito do sistema cofen/conselhos regionais, os
procedimentos para registro de títulos de pós – graduação lato e stricto
sensu concedido a enfermeiros e aprova a lista das especialidades. Diário
Oficial da União.

[7] Barnard J (2012). Remédios Florais de Bach: Forma e Função.

[8] Añael AYT, Pí MG, Castellanos MAG, Gómez DLA, Ortega SMR (2014). MEDISAN.

[9] Guerrini IA, Domene TG (2020). Como as conexões quânticas auxiliam na busca da saúde integral: as bases
científicas da terapia floral e de outras terapias sutis.

[10] Pinto RH, Sousa SM, Santos CR, Senna SM, Leal LP, Vasconcelos EMR (2020). Efeito da terapia floral no estresse docente: Ensaio Clínico
Randomizado. Rev Min Enferm..

[11] Botelho SH, Soratto MT (2012). A terapia floral no controle do estresse do professor
enfermeiro. Saúde Rev..

[12] Baccaro A (1998). Vencendo o Estresse: como detectá-lo e superá-lo.

[13] Kurebayashi LF, Turrini RNT, Souza TP, Takiguchi RS, Kuba G, Nagumo MT (2016). Massage and Reiki used to reduce stress and anxiety: Randomized
Clinical Trial. Rev Lat Am Enfermagem..

[14] Luft CDB, Sanches SO, Mazo GZ, Andrade A (2007). Versão brasileira da Escala de Estresse Percebido: tradução e
validação para idosos. Rev Saude Publica..

[15] Brasileiro SV, Orsini MRCA, Cavalcante JA, Bartholomeu D, Montiel JM, Costa PSS (2016). Controversies regarding the psychometric properties of the Brief
COPE: The case of Brazilian-Portuguese version “COPE Breve”. PloS One..

[16] Cohen J (1988). Statistical power analysis for the behavioral sciences.

[17] Nunes MFO, Hutz CS (2014). Análise da produção de artigos científicos sobre o lazer: uma
revisão. Psicol Teor Pesq..

[18] Bublitz S, Guido LA, Kirchhof RS, Neves ET, Lopes LFD (2015). Sociodemographic and academic profile of nursing students from
four brazilian institutions. Rev Gaucha Enferm..

[19] Conchas-Cisternas Y, Guzmán-Muñoz E, Valdés-Badilla P, Lira-Cea C, Petermann F, Celis-Morales C (2018). Factores de riesgo asociados a bajo nível de actividad física y
exceso de peso corporal en estudiantes universitarios. Rev Méd Chile..

[20] Rodríguez-Munõz PM, Carmona-Torres JR, Rodríguez-Barrego MA (2020). Influence of tobacco, alcohol consumption, eating habits and
physical activity in nursing students. Rev Lat Am Enfermagem..

[21] Yosetake AL, Camargo IML, Luchesi LB, Gherardi-Donato ECS, Teixeira CAB (2018). Percieved stress in nursing undergraduate
students. SMAD, Rev Eletrônica Saúde Mental Álcool Drog..

[22] Ab Latif R, Mat Nor MZ (2019). Stressors and coping strategies during clinical practice among
diploma nursing students. Malays J Med Sci..

[23] Nebhinani M, Kumar A, Parihar A, Rani R (2020). Stress and coping strategies among undergraduate nursing
Students: A descriptive assessment from Western Rajasthan. Indian J Community Med..

[24] Daniel MAI, Soratoo MT, Ceretta LB, Schwalm MT, Zimmermann KCG, Dagostim VS (2013). A terapia floral no controle do estresse
ocupacional. Rev Saúde Com..

[25] Martín BCR (2012). Esencias florales de Bach: efecto del White Chestnut sobre los
pensamientos intrusos indeseados. Rev Cubana Invest Bioméd..

[26] Benham G (2019). The Sleep Health Index: Correlations with standardized stress and
sleep measures in a predominantly Hispanic college student
population. Sleep Health..

[27] Brouwer KR, Walmsley LA, Parrish EM, McCubbin AK, Welsh JD, Braido CEC (2021). Examining the associations between self-care practices and
psychological distress among nursing students during the COVID-19
pandemic. Nurse Educ Today..

[28] Huang L, Lei W, XU F, Liu H, Yu L (2020). Emotional responses and coping strategies in nurses and nursing
students during COVID-19 outbreak: a comparative study. PloS One..

[29] Fitzgerald A, Konrad S (2021). Transition in learning during COVID-19: Student nurse anxiety,
stress, and resource support. Nurs Forum..

[30] Fonseca JRF, Calache ALSC, Santos MR, Silva RM, Moretto SA (2019). Association of stress factors and depressive symptoms with the
academic performance of nursing students.

